# Inferring MicroRNA Regulation of mRNA with Partially Ordered Samples of Paired Expression Data and Exogenous Prediction Algorithms

**DOI:** 10.1371/journal.pone.0051480

**Published:** 2012-12-19

**Authors:** Brian Godsey, Diane Heiser, Curt Civin

**Affiliations:** 1 Department of Statistics and Probability Theory, Vienna University of Technology, Vienna, Austria; 2 Institute for Genome Sciences, University of Maryland School of Medicine, Baltimore, Maryland, United States of America; 3 Department of Oncology, Sidney Kimmel Comprehensive Cancer Center, Johns Hopkins University School of Medicine, Baltimore, Maryland, United States of America; 4 Center for Stem Cell Biology and Regenerative Medicine, University of Maryland School of Medicine, Baltimore, Maryland, United States of America; 5 Greenebaum Cancer Center, Departments of Physiology and Pediatrics, University of Maryland School of Medicine, Baltimore, Maryland, United States of America; UCLA-DOE Institute for Genomics and Proteomics, United States of America

## Abstract

MicroRNAs (miRs) are known to play an important role in mRNA regulation, often by binding to complementary sequences in “target” mRNAs. Recently, several methods have been developed by which existing sequence-based target predictions can be combined with miR and mRNA expression data to infer true miR-mRNA targeting relationships. It has been shown that the combination of these two approaches gives more reliable results than either by itself. While a few such algorithms give excellent results, none fully addresses expression data sets with a natural ordering of the samples. If the samples in an experiment can be ordered or partially ordered by their expected similarity to one another, such as for time-series or studies of development processes, stages, or types, (e.g. cell type, disease, growth, aging), there are unique opportunities to infer miR-mRNA interactions that may be specific to the underlying processes, and existing methods do not exploit this. We propose an algorithm which specifically addresses [partially] ordered expression data and takes advantage of sample similarities based on the ordering structure. This is done within a Bayesian framework which specifies posterior distributions and therefore statistical significance for each model parameter and latent variable. We apply our model to a previously published expression data set of paired miR and mRNA arrays in five partially ordered conditions, with biological replicates, related to multiple myeloma, and we show how considering potential orderings can improve the inference of miR-mRNA interactions, as measured by existing knowledge about the involved transcripts.

## Introduction

MicroRNAs (miRs) are short RNA sequences which are known to affect expression of messenger RNA (mRNA), often by binding to complementary sequences and either inhibiting translation or directing cleavage of that mRNA. A large database of miR information and annotation can be found at www.mirbase.org 1–3. While much research has been performed on miR-mRNA interactions, it continues to be difficult to infer such interactions in large numbers. Typically, these interactions are validated one at a time, though high-throughput methods have recently been developed in an attempt to speed up the process of miR target discovery. We discuss these methods in the following paragraphs.

Recently, some of the most successful attempts to identify likely target pairs include the integration of expression data–most often microarrays–with sequence-based target prediction algorithms that consider the binding affinities between a particular miR and a complementary or near-complementary section of an mRNA sequence. Each data source by itself is prone to error–expression data are noisy, correlation does not imply causation, and prediction algorithms are rife with “false” positives. But, the combination of information from two very different sources had led to vast improvements in the ability to identify likely candidate target pairs. A nice review of the topic can be found in [4].

Most algorithms that combine target predictions with expression data require such data for both miRs and mRNA, but even when miR expression data are unavailable, it is possible to infer miR activity and effective regulation under various experimental conditions using gene expression data and calculated binding strengths from target prediction algorithms [5].

When miR expression data is available, *GenMiR++* [6], one of the first algorithms to combine sequence-based prediction and expression data for both miR and mRNA to infer interactions, uses variational Bayesian methods to infer negative (down-regulating) interactions in expression data, given an 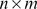 binary matrix where a 1 in entry 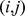 indicates that miR 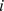 is predicted to target mRNA 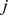. This algorithm was later updated, to become, eventually, *GenMiR3* [7], with the most prominent update being that the newer algorithm is able consider sequence-based information (not just presence or absence on a list of predictions) in determining the strength and likelihood of targeting interactions.

*TaLasso* [8] is another prominent algorithm that combines the presence of an miR-mRNA pair in a targeting prediction database with expression data. It uses LASSO regression, restricted to non-positive interactions, and includes tuning parameters to adjust the sensitivity/sparseness of the solution. *TaLasso* has been shown to outperform *GenMiR++* in some cases [4], [8].

Another Bayesian model proposed by Stingo, *et al* [9], uses a Markov chain Monte Carlo (MCMC) algorithm to fit the model and estimate parameter values, using a model formulation that is similar to that of *GenMiR++* and *GenMiR3*. It restricts interactions to be non-positive using a combination of binomial and gamma distributions, like both *GenMiR* algorithms, and can include sequence-based algorithms and scores, as *GenMiR3* does. In addition, a “time-variant” version of the model is presented, in which targeting parameters are allowed to vary over time in a time-series data set.

In some cases, a basic Pearson correlation is used to rank putative targets, possibly in combination with prediction algorithms [4], [10]. Spearman correlation and other varieties of regression have been proposed for the same task, but none have performed as well as *GenMiR* or *TaLasso* [4].

In this paper, we propose a Bayesian model for inferring miR-mRNA targeting interactions based on target prediction algorithms and expression data, which we fit using variational Bayesian methods like the *GenMiR* algorithms, and which can utilize any sequence-based (or other external) information like *GenMiR3* and the Bayesian model from Stingo, *et al*.

However, in contrast to one or more of the aforementioned algorithms, our model:

1. considers both positive and negative interactions between miR and mRNA.
2. uses a normal distribution to characterize interaction strength.
3. optimizes the weights/coefficients placed on sequence/prediction information via the same variational Bayesian algorithm that estimates the rest of the model parameters.
4. accounts for data replicates, biological or technical, and propagates uncertainty throughout the model parameter estimates.
5. can consider a partial ordering of the samples.

With respect to these points, we enumerate how the three algorithms described–*GenMiR3*, *TaLasso*, and the Stingo model–differ from our model:

1. All three algorithms consider only negative interactions, but we chose to consider both positive and negative interactions since some positive indirect effects may, in some cases, better explain changes in expression values than negative effects only [11], [12]. We still have the option, when searching for direct miR targets, to consider only the inferred negative interactions; we explore this option in the *Results* section.
2. We chose to use a normal distribution to characterize the interaction coefficients where *GenMiR3* and the Stingo model have used combined binomial and gamma distributions. The binomial-gamma combination more strongly enforces sparseness in interactions, but considers only negative interactions, as mentioned. *TaLasso* is non-Bayesian and provides no distribution for these coefficients.
3. Both our model and the Stingo model estimate the influence of external target prediction information in the same manner as other parameters (variational Bayes and MCMC, respectively) while *GenMiR3* uses the [non-Bayesian] conjugate-gradient method to optimize the weights placed on the target prediction information. *TaLasso* doesn’t consider such information.
4. Based on their descriptions and implementations, none of the algorithms explicitly account for technical/replicate variance or otherwise allow for grouping of samples without taking their average value before starting the algorithm.
5. With the exception of the Stingo model, which in its “time-variant”version allows some interaction parameters to change over time, none of the models considers an ordering of the expression samples.

In the following sections, we specify our model and demonstrate its ability to reliably infer miR-mRNA interactions in an expression data set of samples taken from multiple myeloma patients in different stages of the disease. We use the *miRWalk* [13] database of validated targets and compare our results with those obtained from *TaLasso*, as well as Pearson correlation, as a benchmark. Throughout, we illustrate how the natural partial ordering of the data can be used to improve interaction inference, particularly if we are concerned mainly with interactions specific to the progression of multiple myeloma development.

## Methods

We have developed a Bayesian model of miR-mRNA interactions for matched expression data (*i.e.* we have both miR and mRNA expression data for each biological sample) that was designed specifically for partially ordered samples, where “partially ordered” refers to the case where every sample can be said to be “before” or “after” at least one other sample in the data set. The partial ordering could indeed be a time-course experiment (which is usually fully ordered, linearly in time) or it could comprise multiple branches of experimental development, such as a disease study wherein healthy and diseased samples–possibly originally all starting from the same healthy population–are collected over time, or in stages.

We have developed a model for partially ordered samples because prior work in using expression data to infer miR-mRNA targeting interactions has focused on methods that do not depend on the order of the samples, the most common of which is Pearson correlation. We find it both theoretically and practically desireable to consider an ordering of samples because in most cases we expect that samples whose sources are more similar–in this case by disease type or stage–should also have the most similar expression values. If we consider such a natural ordering, we should be more likely to infer significant targeting interactions that occur from one stage to the next but whose expression levels are not necessarily the most correlated throughout the entire data set.

Let us consider a simple example of how using an ordering of data can help infer interaction coefficients. Assume a fully-ordered data set of 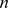 stages 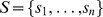, and we have [correctly] inferred the mean log expression values 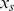 and 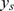 in stage 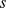 for a single miR 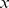 and mRNA 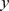, which are perfectly negatively correlated and where each has been normalized to have mean zero and standard deviation of one. A simple model formulation for each 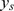, without considering the ordering, could be
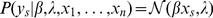
(1)for interaction coefficient 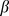 and precision (inverse variance) 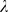. If we use a non-informative but improper uniform prior distribution for 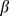
–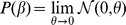
–then the variational Bayesian estimates for 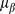 and 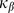 are 
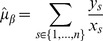
(2)

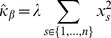
(3)and likewise if we consider the ordering of samples such that for 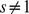,
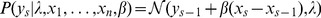
(4)the corresponding estimates are 
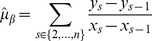
(5)

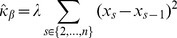
(6)

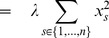
(7)

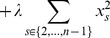
(8)

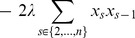
(9)


With the assumed perfect negative correlation and unit standard deviation, both estimates (2) and (5) for 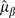 give a [correct] value of 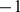. Also, equation (3) is the same as line (7); therefore lines (8) and (9) give the adjustments to the estimated precision of 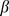 in the ordered model version as compared to the standard version. If the sum of these is positive, the estimate for 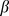 given by the ordered model has a higher precision and thus is more statisically significant. This occurs, for example, in the simple case where 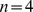, 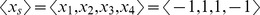 and 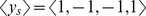, where the ordered precision estimate is 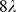 while the unordered one is 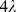. In contrast, if we take a different ordering of the same paired data, 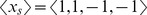 and 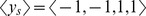, both versions of the model give a precision estimate of 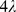. If one thinks sequentially about the data, it seems that in the former example, the miR and mRNA make two simultaneous but opposing expression changes, one between stages 1 and 2 and another between stages 3 and 4. In the latter example, only one such simultaneous change is made. In many cases of miR-mRNA interaction inference, it would be desireable to make a distinction between these two cases, particularly in experiments designed to measure stage-by-stage development of a process, where we might expect the expression levels of an miR to rise for some specific period of the process and then fall again.

We can also generalize a bit from these simple examples. Since the summation in (8) is an approximation of the variance of the 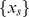 (without the first and last stages), which we have normalized to 1, and the summation in (9) is the [lag 1] autocorrelation of the 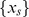, generally speaking, the ordered model gives higher precision for interaction coefficients when the autocorrelation of the normalized expression data is less than 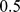. This is not an exact rule since (8) does not include the first or last stages, but we can see that it does not take an unreasonable amount of expression variation between adjacent stages for the ordered model to give higher statistical significance for interaction coefficients, when such an ordering exists.

In addition the possibility of higher statistical significance in highly varying miR, considering the order of samples in an experiment allows us to detect positive or negative trends in expression value with respect to the process being investigated–a feature that may prove useful in identifying the main drivers of a developmental process such as disease, growth, aging, etc. The model also includes scores from existing prediction algorithms for miR-mRNA targeting to better determine the existence of a targeting interaction. In this paper, we have used data (including prediction scores) from the *TargetScan* [14]–[17] and *miRanda* [18], [19] databases, but any exogenous, quantitative information about the putative target pair could be included.

Below, we define and fit our model to a previously published multiple myeloma data set using variational Bayesian methods. Then, we check our results against the *MiRWalk* [13] database of experimentally-validated targeting interactions, as well as against rankings of predicted target pairs by most negative Pearson correlation coefficient.

### Data

We demonstrate our model using the multiple myeloma data set from [20], which can be downloaded from *GEO* [21], accession number GSE17498. In this data set, there are both miR (Agilent-019118 Human miRNA Microarray 2.0) and mRNA (Affymetrix Human Genome U133A Array) expression values for samples from 36 patients, 34 of whom have been diagnosed with multiple myeloma (MM) and 2 of whom have been diagnosed with plasma cell leukemia (PCL). Unfortunately, from healthy donors there is only miR expression data and no mRNA data, so we cannot include healthy samples in our study. However, the diseased samples can be arranged into four Durie-Salmon stages: IA, IIA, IIIA, and IIIB, which gives an obvious ordering for our non-PCL samples. Because PCL is a condition related closely to, but not necessarily developing directly from (or progressing to) MM, we treat it as a separate branch of the partial ordering, as described in the subsequent section. However, within our partial-ordering framework it is necessary to specify some relation to other samples, and thus we assume that PCL follows the healthiest, most normal MM stage–in the absence of truly healthy samples–Durie-Salmon IA.

### Partial Ordering

**Figure 1:**
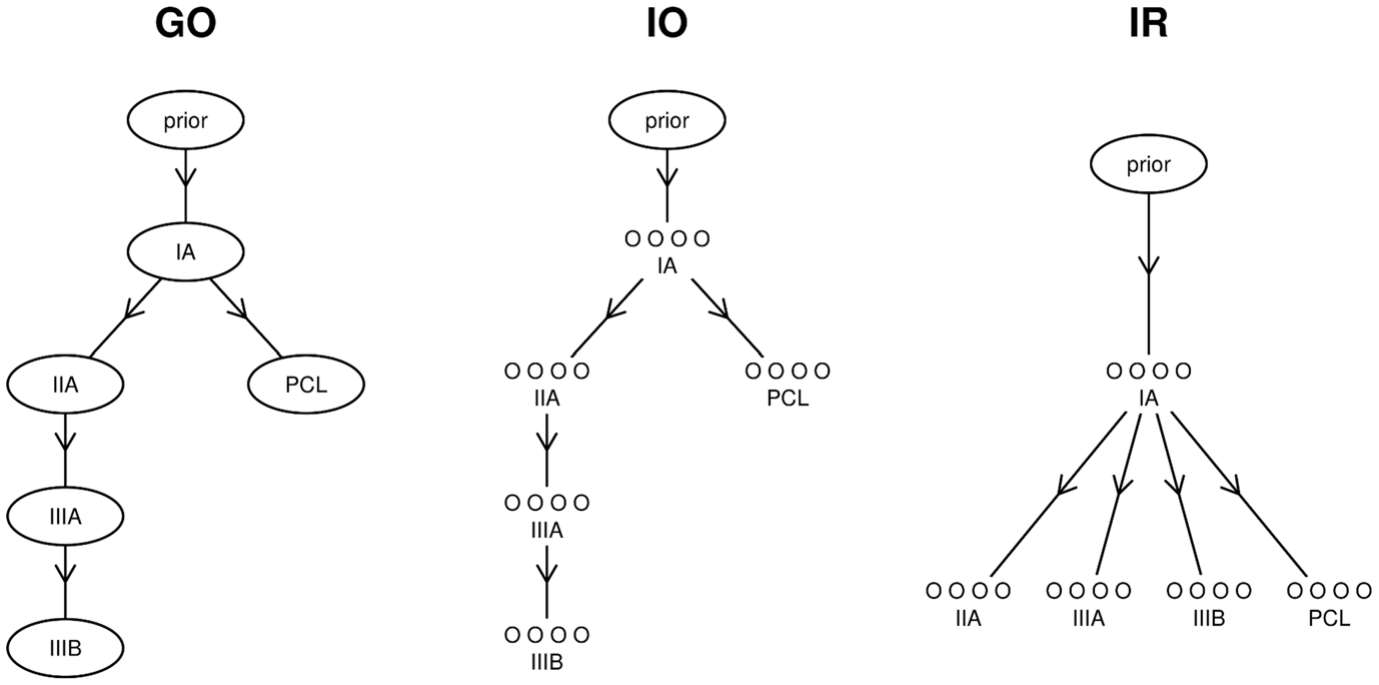
Partial orderings. The graphs above show the three different partial orderings of the data that we explore in this paper. Arrows give the direction of the ordering, where sample A can be said to *precede* sample B (i.e. 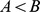) if in the graph an arrow points from A to B. *G-O* refers to the grouped-ordered model version, in which samples of the same Durie-Salmon stage are grouped together as replicates. *I-O* is the individual-ordered model version, where the groups of smaller circles represent that different samples are not grouped as replicates, but the ordering of Durie-Salmon stages is the same as in the *G-O* ordering (i.e. 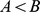 if and only if the stage of A precedes the stage of B in the *G-O* ordering). And, *I-R* is the individual-reference model version, where the samples are again not grouped as replicates, but each of the Durie-Salmon stage IA samples precedes each sample of every other stage. In each partial ordering, there is a prior distribution over the samples which are not preceded by any other samples.

In our analyses, we compare three different partial orderings, which we show in Figure 1. The first is the natural ordering: the Durie-Salmon stages in order, plus PCL as a separate branch off of the initial stage, stage IA. This ordering treats patients with the same disease type and stage as replicates, and should give results that are specific to the disease itself since it effectively ignores the differences between individuals with the same disease type. We call this ordering the grouped-ordered (*G-O*) ordering.

The second partial ordering is the same as the first, but with the individuals separated. We call this the individual-ordered (*I-O*) ordering. In this version of the model, each individual comes after all of the individuals of the prior Durie-Salmon stage, and again the PCL samples are after all of the IA samples. This arrangement places less focus on the disease itself but allows the model to infer miR-mRNA interactions based on variations between individuals of the same type.

The third partial ordering considers the stage IA samples to be references, while all other samples come directly after them. Individuals are still considered separately. Hence, this is called the individual-reference (*I-R*) ordering. This reference-based design largely ignores the natural ordering of the data and focuses on differences between individuals. Comparing the results from this ordering with the results from the other orderings could indicate some of the advantages (and disadvantages) of considering the natural ordering of these samples.

### Pre-processing

Prior to the main analysis, we performed quantile normalization across all arrays of the data set using the *limma* package for *R* [22], [23]. We then performed a probewise ANOVA to test for differential expression across the stages IA, IIA, IIIA, IIIB, and PCL (using individuals within a stage as replicates) and removed those probes/probesets (for both miR and mRNA) whose (unadjusted) p-value from the ANOVA F-test was greater than or equal to 0.05, as well as those miRs and mRNAs not involved in any predicted targeting interactions, leaving 28 miRs and 367 mRNAs as possible candidates for targeting interaction. Lastly, we re-scaled the data so that each probe/set–across all samples–had a mean of zero and a standard deviation of one.

### Target Prediction Algorithms

For these analyses, we included miR-mRNA target prediction data from both *TargetScan* [14]–[17] and *MiRanda* [18], [19]. For each of these, we downloaded from the corresponding web site a table of predicted miR-mRNA interactions and the targeting scores calculated by the respective algorithms. *TargetScan* includes a *context+* score and *MiRanda* includes a *mirSVR* score [24]. In our model, described below, we consider the predicted interactions and these prediction scores.

### Model

Let us define a stage as a set of expression levels 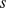 that are, for each probe/set, to be considered replicates of each other. We assume a partial ordering 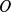 on the set of stages 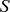 such that each stage 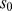 has a set of parent stages 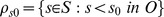. The set of parent stages for 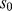 can be defined as 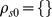 if 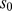 has no parents and is therefore an initial stage in 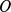 (the existence of at least one initial stage is guaranteed by the acyclic property of partial orderings). Furthermore, let us define a “development” parameter 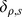, which intuitively represents a kind of distance from a parent 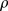 to its child 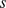, and which we include in the equations below. Likewise, let 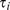 be the “trend” of probe/set 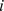 in either the positive or negative direction with respect to the ordering 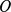, and let 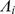 be the precision (inverse of variance) parameter of the expression of probe/set 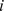 thoughout all stages.

#### miR parameters

Then, we assume the log expression value 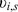 of each miR 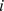 in stage 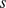, to be normally distributed with mean
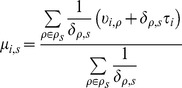
(10)and precision



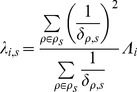
(11)Thus, the prior mean 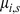 is the weighted sum of the parents’ expression values with the developmental trend added (the trend 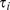 multiplied by the development factor 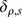). The weights in the weighted sum are the inverses of the developments 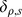. Likewise, the prior precision is the weighted average of inverse developments multiplied by the probe’s stage-wise precision parameter 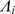. This formulation gives two parents equal weight in the prior distribution of a common child 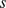 if their development parameters to that child, 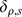, are equal. Also, as 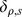 increases for one parent to the child, the influence of its expression value on the child’s prior distribution diminishes to zero.

Note that in the formula for the stage’s prior precision 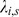, the probewise precision 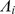 is moderated by 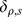, in that a parent stage 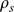 that is more similar to its child 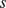
–if it has a smaller value 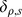
–carries more weight and increases the precision in the prior distribution of the probes in stage 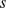. This allows for varying developmental distances between stages, where larger 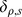 imply that all probes experience lower precision (more random noise) between stages 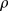 and 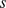, and vice-versa for smaller 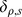.

#### mRNA parameters

The distributions we have assumed for mRNA expression are identical to those of the miR, except that the developmental trend component (the product 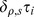) is exchanged for an interaction component with the miR expressions 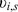. Let 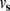 be the vector of all miR expression values in the stage 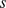, and let 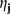 be the vector of interaction coefficients between 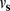 and mRNA expression level 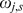, then 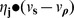
–where 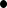 is the standard vector dot product–expresses the total interaction effect of all miRs on the mRNA 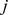 from stage 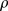 to stage 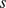.

Specifically, we assume the log expression value 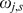 of each mRNA 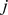 in stage 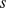, to be normally distributed with mean
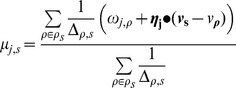
(12)


and precision
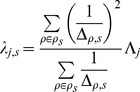
(13)where the 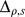 are analagous to, but distinct from, the 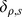 we used in the miR distributions. Likewise, the 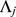 are analagous to the 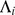 from the miR distributions, but are inferred separately for each mRNA 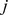, as they are for each miR 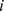.

#### Technical and replicate variance

We assume two technical precisions (inverse variances) in our model. One precision corresponds to an expression set (i.e. the precision/variance between microarrays from the same stage) and one corresponds to replicates within one expression set (i.e. multiple spots for the same probe/set or transcript within a microarray).

For the miR and mRNA expression levels, 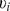 and 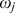, above, we assume that the expression levels 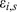 and 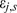 each probe 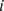 (miR) or probeset 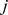 (mRNA) in an expression set 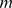 from stage 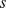 is normally distributed as
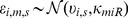
(14)or
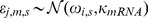
(15)where the second parameter 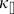 in the normal distribution 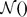 is a precision, not a variance or standard deviation.

Furthermore, within each expression set 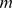, we assume that the expression data 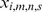 (miR) and 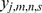 (mRNA) for within-set replicate 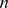 and stage 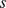 are normally distributed as
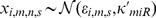
(16)or
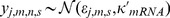
(17)for the two second-level technical precisions 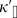.

#### Interaction parameters

Each element 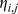 of the vector of interaction coefficients 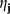 metioned above is also normally distributed as
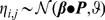
(18)where 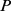 is the vector of fixed parameters from target prediction algorithms, 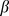 is a vector of estimated coefficients, and 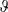 is again a precision. Note that there is no restriction of the interaction coefficients 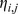 to only negative values, as in some models, as we choose to allow for all regulatory effects, positive or negative, direct or indirect.

If one of the included algorithms predicts that miR 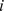 targets mRNA 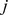, we include the vector 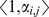 where 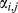 is the prediction score from the algorithm. We concatenate the vectors from multiple prediction algorithms such that, if a pair 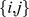 is predicted by more than one algorithm, the vector 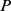 is of the form 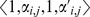. In this way, the coefficients of 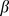 that correspond to a 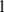 in 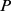 determine the effect that inclusion on a particular list of predicted targets has on the expression data (indirectly through estimation of 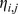), while the coefficients corresponding to algorithm scores may further refine the value of the algorithm in this model. (Also, an algorithm score of zero does not necessarily indicate zero chance of targeting, and thus if we include the scores, we must also include a constant.).

#### Prior distributions

We chose conjugate prior distributions for each model parameter that required a prior. Thus, we use vaguely informative normal distributions on the parameters 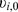, 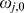, and 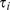. Specifically, they all follow the distribution 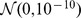. Similarly, the prior distribution for 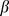 is the equivalent multivariate normal with zero mean and precision matrix 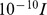, where **I** is the identity matrix of the appropriate size. We use vaguely informative gamma prior distributions on the parameters 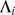, 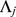, 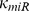, 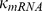, 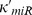, 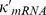, and *φ*.

The development parameters 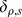 and 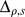 are special cases. Foremost, they have no obvious conjugate priors, and as we have defined this model, their optimal values are not unique since, for example, doubling the estimated values for 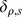 and 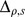 along with the 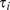 would give an identical likelihood, if priors are ignored. However, we are not concerned with the specific values of these parameters; we need only their values relative to each other. Thus, in order to obtain unique optimal values, we specify a gamma prior on the 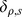 and 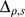 with shape and rate (i.e. inverse-scale) set equal to 1.

### Fitting the Model using Variational Bayes Methods

To estimate the parameters of our model, we use variational Bayesian methods. These methods are closely related to expectation-maximization algorithms [25] and have been used previously in discovering miR-mRNA target pairs [6], [7], [26] as well as other analyses of gene expression data [27], [28].

In short, variational Bayesian methods find a probability distribution 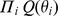 (factorizable over all parameters) that is increasingly similar (via iterative updates) to the desired posterior distribution 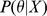 for model parameters 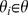 and data 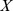, through use of the Kullback-Leibler divergence as a measure of dissimilarity. As part of the calculations, one also obtains a lower bound 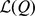 to the *evidence* 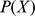, which can be helpful in judging the goodness of fit of alterative model formulations. For a thorough explanation of variational Bayesian methods, see [29] or [30].

The result of variational Bayesian calculations is, like with most Bayesian methods, a set of estimated posterior probability distributions over the model parameters. Unlike Markov chain Monte Carlo and related methods, we need not worry much about convergence of the estimated parameter distributions, since, if implemented properly, a variational Bayesian algorithm guarantees an improvement in every iterative update. Of course, calculations can be quite slow when compared to non-Bayesian methods.

All parameters were estimated using variational Bayesian (VB) methods, with the exception of the 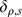 and 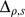, since they have no simple conjugate distributions. Specifically, we treat the 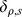 and 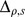 as fixed values while iteratively updating the other parameters, and then we update these by maximizing the lower bound 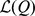 over their possible value range. More specifically, after first initializing the variables to reasonable values, the algorithm sequentially updates the posterior distribution estimate for each variable (excepting 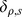 and 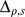) and that posterior estimate (of the same form as the conjugate prior) is utilized in all subsequent steps. After all such posterior estimates have been updated, 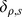 and 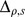 are optimized by finding the maximum likelihood estimates. Then, once again, the posterior distribution estimates are updated for all other parameters, and so on. This process is repeated until the parameter values change very little with each subsequent iteration and thus it becomes no longer beneficial to continue updates. We found that 200 such iterations gave sufficient results.

This algorithm was coded in the *R* [23] statistical programming language.

## Results

We applied our model to the multiple myeloma data set from [20] in three different configurations based on our choice of partial orderings (shown in Figure 1). For these analyses, we consider only those interactions which were predicted by at least one prediction algorithm (*TargetScan* or *miRanda*), but we would like to note that this is not necessary in our framework. We have limited the set of candidate interactions in this way because the high number of possible parameters in the model (all possible interactions) can be significantly reduced by considering only predicted interactions. Furthermore, target predictions are known to have significant significant sensitivity [31], when compared to validated targets, even if they have unknown specificity since a complete list of true targets does not exist. Thus, we attempt to rank only the 1754 interactions predicted between the 28 miRs and 367 mRNAs, ensuring that our top candidate interactions have been predicted as well as supported by expression data.

Below, we evaluate the results and compare them with the interactions rankings one obtains from *TaLasso* as well as by ranking by Pearson correlation coefficient, as a simple benchmark. For this, we consider both the strongest absolute value correlations as well as the strongest negative correlation, as much evidence indicates that miR-mRNA interactions are predominantly negative, and thus ranking by most negative correlation generally improves results [4]. In each of the rankings based on our model, all of the top 900+ inferred interactions were negative; thus, restricting only to negative interactions has no effect on these results.

First, we checked the *miRWalk* database for target pairs that have been experimentally validated and we looked at their ranking according to each of the methods. Second, we looked for enrichment of *KEGG* pathway [32] annotation among mRNAs involved in the top 100 targeting interactions on our lists. To do this, we used the “singular annotations” from the *GeneCoDis* web tool [33], [34]. Then, we examined more closely specific target pairs near the very top of our rankings. Lastly, we looked at the miRs with the strongest trends through the stages of multiple myeloma according to the *G–O* ordering, which we hypothesize indicates a high likelihood of playing a role in the development of the disease.

### Interaction Validation by *miRWalk*

Among our data set, only five putative miR-mRNA targeting interactions have been validated, according to the validation database *miRWalk* [13], though 13 more target pairs have been validated despite not being predicted by either *TargetScan* or *miRanda*. This may indicate that heavily favoring predicted pairs over non-predicted pairs detracts from the results more than expected. However, since one of our main goals here was to combine prediction data and expression data, we do not address the issue here.

All five of the validated, predicted target pairs involve the well-studied miR-17. These five interactions appear at positions 63, 229, 234, 273, and 612 on our interaction ranking based on the *G–O* ordering from Figure 1. This is considerably better than in the ranking by absolute value Pearson correlation (341, 402, 819, 877, and 893) and also by most negative Pearson correlation (162, 195, 468, 568, and 604). The *TaLasso* results gave rankings for only four of these five validated target pairs, as the *TargetScan*-predicted pair {hsa-miR-17, PKD1} seems to be missing; perhaps the results list was truncated or the pair was missing from the built-in list of predictions. However, the remaining four interactions are ranked 311, 351, 846, and 952, which is comparable to Pearson correlation.

If we divide our ranking positions, in increasing order, by the rankings by correlation (*e.g.* we divide 63 by 341, 229 by 402, and so on), we find that our rankings are, on average, 0.41 times those by absolute value correlation and 0.71 those by negative correlation. Repeating this using the four rankings from *TaLasso* and the top four from our model gives 0.35. This can be interpreted as an estimate of the relative number of target pairs that would need to be experimentally tested based on each ranking in order to arrive at the same number of positive validations, and from now on we will refer to this statistic as the “average relative rank statistic”. If we consider our ranking using the partial ordering *I-O* from Figure 1, we obtain average relative rank statistics of 0.39 and 0.56 when compared to rankings by absolute value correlation and negative correlation, respectively. Partial ordering *I–R* gives average relative rank statistics nearly identical to these.

Though there are too few existing validations for us to draw strong conclusions, the fact that the rankings of these by our model are much closer to the top of the list than those by the correlation (negative or absolute value) indicates that there is at least some advantage to our partially ordered model.

### KEGG Pathway Enrichment

We show in Table 2 all of the KEGG pathways that are enriched (FDR corrected 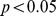) in the top-100 list for at least one of the four models (including ranking by negative correlation among predicted target pairs). There are considerably more pathways with enrichment among the lists generated by our model than by negative Pearson correlation. Specifically, six types of cancer appear in the lists for our models, while there are none for the correlation list. This is promising, as this data set comes from a cancer study, specifically of multiple myeloma. In total, among the 41 genes involved in the top 100 interactions inferred by the *G–O* ordering, there were 13 enriched pathways, and only two among the 56 genes in the top 100 interactions according to ranking by negative correlation.

**Table 2:** Enriched KEGG pathways among genes in the top 100 interactions.

	G–O	I–O	I–R	TaLasso	Neg.Cor
Number of unique genes in the top 100 interactions	41	58	53	85	56
05200 :Pathways in cancer	3				
05215 :Prostate cancer	2	3	3		
05219 :Bladder cancer	2	2	2		
05222 :Small cell lung cancer	2		2		
05216 :Thyroid cancer	2				
05214 :Glioma		2	2		
05218 :Melanoma		2	2		
05016 :Huntington’s disease	3	4	3		
05014 :Amyotrophic lateral sclerosis (ALS)	2	2	2		
05010 :Alzheimer’s disease		3			
04976 :Bile secretion		2			
04730 :Long-term depression			2		
04115 :p53 signaling pathway	2	3	4		
04210 :Apoptosis	2	2	2		
04010 :MAPK signaling pathway	3		3		
04722 :Neurotrophin signaling pathway	2				
04110 :Cell cycle	2				
04120 :Ubiquitin mediated proteolysis	2			4	
04622 :RIG-I-like receptor signaling pathway		2	2		
04144 :Endocytosis				4	
04914 :Progesterone-mediated oocyte maturation				3	
04114 :Oocyte meiosis				3	
04142 :Lysosome				3	
03060 :Protein export					3
04141 :Protein processing in endoplasmic reticulum					4

The top row gives the number of unique genes present in the top 100 miR-mRNA interactions according to each model; the remaining rows give, per column, the number of these genes annotated by *KEGG* pathway terms with significant enrichment (FDR corrected 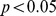) for for at least one of the models proposed. A blank entry indicates that the particular pathway was not significantly enriched in the model. The column *G–O* refers to the grouped-ordered model version, *I–O* is the individual-ordered model version, and *I-R* is the individual-reference model version, while *Neg.Cor* is the ranking by most negative Pearson correlation (between miR and mRNA expression profiles) among the predicted target pairs. The horizontal lines separate general categories of *KEGG* pathways, namely cancer-related pathways, other disease-related pathways, and then remaining pathways found to be enriched by at least one of the models.

Amongst the three partial orderings we have considered here, there is marginally more pathway enrichment and fewer genes involved in the top 100 targeting interactions for the *G–O* ordering, though both the *I–O* and *I–R* orderings both give much more enrichment than the rankings by *TaLasso* and negative correlation.

### Top Candidate Interactions

**Figure 2:**
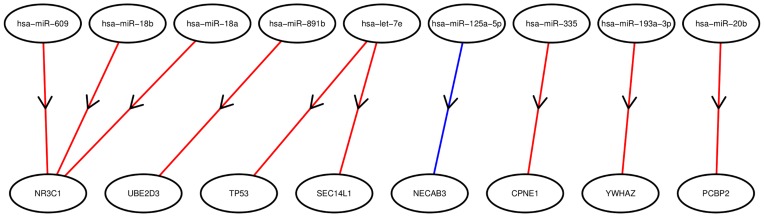
The top 10 interactions according to the *G–O* ordering. In the above diagram, we show the miRs (top row) and genes (bottom row) involved in the 10 most significant targeting interactions based on the *G–O* ordering from Figure 1. In each case, the inferred interaction is negative, meaning that the miR inhibits the expression of the corresponding gene. A red line from an miR to an mRNA indicates that the interaction was predicted by *TargetScan* and a blue line indicates that the interaction was predicted by *miRanda*.

Ultimately, our goal with this analysis is to enable the identification of the most promising candidates for further biological investigation. In Figure 2 we show the top ten interactions inferred by the model using the *G–O* ordering. The three strongest inferred interactions involve the NR3C1 glucocorticoid receptor, which first appears in the 109th interaction on a ranking of interactions by negative correlation. Myeloma patients with low expression of this receptor respond poorly to standard treatment with dexamethasone and have a poor overall prognosis, making this molecule an intrinsically interesting candidate for further investigation [35]. Two of the miRs inferred as targeting this gene, miR-18a and miR-18b (part of the 5th and 6th ranked interactions by negative correlation), share a seed sequence, and are associated with the miR-17
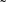
92 cluster–a downstream target of the c-myc oncogene [36]. This cluster is well-known to play a role in cancer development as well as normal lymphoid development, and has recently been associated with tumorgenicity in multiple myeloma [37]. The next strongest inferred interaction involves the gene UBE2D3, (targeted by miR-891b) which is a ubiquitin-conjugating enzyme known to be involved in p53 ubiquitination [38]. The next ranked interaction on our list involves the p53 tumor-suppressor (TP53)–an extremely important gene in most, if not all, cancer types–inferred to be targeted by miR-let-7e. Unlike in many cancers, at diagnosis in multiple myeloma, p53 is rarely seen to be mutated or deleted. As it is not changed at the genomic level, it is therefore quite plausible that p53 may be manipulated at the level of translation by miR in this disease, making this pair an intriguing candidate interaction as well.

### Inferred miR Regulators in Multiple Myeloma Development

The inclusion of trend parameter 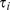 for each miR in our model allows us to identify miRs whose expression levels increase or decrease significantly over the progression of stages with respect to the partial ordering. Table 1 shows the miRs with a corresponding trend parameter 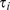 estimate whose posterior mean is more than three standard deviations (based on the posterior precision estimate) from zero, in either direction, positive or negative.

**Table 1:** miRs with a significant estimate for the trend parameter.

trending miR	direction	z-score
miR-18b	+	3.88
miR-367	−	3.80
miR-18a	+	3.61
miR-194	+	3.57
miR-133b	+	3.54
miR-92a	+	3.38
miR-554	−	3.24
miR-551a	+	3.23

Shown are the most significant trend parameter estimates (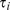). A “+” in the table denotes that the expression of the miR increased throughout the progression of the partial ordering by disease stage (*G–O*), and tended to be higher during later stages. Likewise, a “−” denotes that the expression of that miR tended to be higher in early stages and lower in later stages.

Top candidates from the table include miR-18a and miR-18b, which, as discussed above, are well-known to play a role in cancer development. Both of these showed increased expression in advanced stages of multiple myeloma. Another candidate is miR-194, which has been shown to be p53-dependent and a positive regulator of this well-known tumor-supressor, creating a positive feedback loop. Furthermore, down-regulation of miR-194 has been demonstrated to play a key role in multiple myeloma development through its modulation of p53 signaling [39]. Our model inferred a significant positive trend for miR-194, which might be contrary to this prior expectation of down-regulation, but in any case adds to the evidence that miR-194 is involved–perhaps in a complex way–in the development of multiple myeloma.

## Discussion

Combining miR-mRNA target prediction algorithms with expression data has proven to be one of the best strategies for high-throughput target pair inference. However, the exact way in which do this has been the subject of some discussion. Though many methods have addressed specific issues in target inference, and others have attempted a more general approach, none has fully addressed ordered and partially ordered data sets. We tried three different partial orderings in our model, as shown in Figure 1. They performed similarly to each other, but not quite the same, supporting the conclusion that the ordering does make a difference, and thus should be carefully considered before analysis. Grouping and ordering samples by disease stage seems to have enriched, if only slightly, the top target interactions according to our *KEGG* anaylsis.

As illustrated in the *Methods* section, the order of samples (if one exists) can affect the strength of inference of correlated miR and mRNA expression patterns, and in fact this additional statistical power can be seen in a very simple example. The model that we present in this paper addresses partially ordered data sets by assuming that closely related samples (with respect to the ordering) should be more similar than less closely related samples. This assumption allows the model to outperform *TaLasso* and Pearson correlation (*i.e.* ranking of target pairs by most negative correlation) by a noticeable margin, aided by the Bayesian framework that inherently places more weight on measurements and variables that have high certainty or precision. Our model’s rankings of the few previously experimentally validated target pairs were significantly better in our model, and KEGG pathways were significantly more enriched, particularly for cancer-related pathways, which we would expect from this data set.

Both the mRNA targets and targeting miRs from our top-ranked interactions have been previously implicated in multiple myeloma development, suggesting that our analysis has successfully identified biologically-relevant pairs from this data set. Furthermore, some of the miRs that we have identified as having a significant trend through the ordering of the stages have been verified by literature as key players in both cancer and, more specifically, multiple myeloma. The remaining un-verified top interactions and trending miRs may be good candidates for further investigation.

Interestingly, though we didn’t limit our interactions to be non-positive, virtually all of the top 1000 interactions were negative. This is likely an effect of utilizing the the prediction algorithms in the prior distributions for the interaction parameters, since our model estimates coefficients for the inclusion in (and targeting score of) each included prediction algorithm. It is well known that miRs typically down-regulate target mRNAs, and though there have been some reports of up-regulation, we would expect the estimated coefficients for predicted targets would lead to a negative prior distribution (see equation 18) on the majority of interactions, if not all of them.

One potential weakness of our model–which is shared by virtually all recent models of miR-mRNA targeting–is that we attempt to explain all changes in mRNA expression using miR targeting interaction coefficients. This assumption that miR targeting should account for all gene expression changes is patently untrue. There are other direct processes–involving transcription factors, for instance–as well as indirect processes that can affect mRNA expression. Though it would be quite cumbersome in both data and calculation, an expanded model taking into account other potential influences could prove very useful in inferring true interactions between the various nucleic acids, proteins, etc.

Lastly, though much literature has been published on the topic, we have a lot to learn about high-throughput inference of miR-mRNA target pairs. Experimental validations are so sparse that it is impossible to prove conclusively which prediction or inference techniques routinely give the best results, and in which cases each is most appropriate. Perhaps in the near future we will see a vast increase in the number of targets being validated, possibly through cooperation or organization between research groups to create more complete databases (both of positive and negative results) with which we can compare inference approaches to further refine our methods and in turn more efficiently focus our experimental efforts into the most promising areas.
